# Purification of endoglucanase produced by *Penicillium citrinum *isolated from Amazon

**DOI:** 10.1186/1753-6561-8-S4-P221

**Published:** 2014-10-01

**Authors:** Anita Lima de Souza, Pamella Suely Santa Rosa Pimentel, Edmar Vaz de Andrade, Spartaco Astolfi-Filho, Carlos Gustavo Nunes-Silva

**Affiliations:** 1 Biotech Amazonia LTDA-ME - BIOTECH AM, Manaus, Brazil; 2 Universidade Federal do Amazonas, UFAM, Manaus, AM, Brazil

## Background

The cellulolytic enzyme complex has many important biotechnological applications such as beverage, textile, food, paper and cellulose industries, as well as in degradation of lignocellulosic for ethanol biofuel production [1]. According to the enzymatic activity, cellulolytic complex is subdivided in three classes: endoglucanases, exoglucanases and β-D-glycosidases.

Endoglucanases (endo-β-1,4-glucanase, EC 3.2.1.4) are responsible to initiate cleavage, hydrolyzing randomly internal regions from the amorphous cellulose fiber structures, releasing oligosaccharides from different grades of polymerization. These will be hydrolyzed by exoglucanases releasing cellobiose followed by β-D-glycosidades being hydrolyzed to glucose [1].

*Penicillium citrinum *has worldwide occurrence. Commonly in the soil, this specie is described as good xylanase and cellulase producer [2,3]. This work describes liquid chromatography to separate endoglucanases of *Penicillium citrinum *supernatant from submerse fermentation process.

## Methods

Suspension of 1 mL *P. citrinum *spores (6.4 × 10^6^) was inoculated in 200 mL submerse fermentation medium [4] containing CMC as carbon source for 10 days/28°C/150 rpm. Every 24 hours was sampled 6 mL aliquots, centrifuged and right after the supernatant storage at 4°C. A pool was made of collected points from 72 to 240 hours. It was concentrated in SpeedVac (Thermo) for final volume of 1.5 mL, filtrate (0.45 µm) and injected in chromatograph column Superdex 75 10/300 GL (GE Healthcare) previously equilibrated with sodium citrate buffer at 50 mM pH 4.8. Fractions of 1.5 mL was collected in a flux of 0.6 mL/min in 1.5 column volumes (CV) using Akta Purifier (GE Healthcare). Fractions were analyzed by CMCase activity [5], protein dosage with BCA kit (Thermo Scientific) and SDS-PAGE 12%.

## Results and conclusions

It was observed a elution profile with three peaks from purification of 12 mL (pool) from culture supernatant, corresponding to the fractions 7, 8, 11 and 16, containing 60, 60, 250 and 30 µg/mL of proteins, respectively. By analysis in SDS-PAGE a band with 33 kDa in fractions 7 and 8 was detected, matching for endoglucanases of genera Penicillium, which can vary from 26 to 50 kDa. Only fraction 7 and 8 had CMCase activity (0.4 and 0.3 U/mL, respectively), in which 1 unit is the necessary quantity to release 1 µmol/min of hydrolyzed product. The specific activity CMCase was 6.6 and 4.4 U/mg for 7 and 8 fractions, respectively. Chromatographic profile obtained for each induction time (72 to 240 hours) was comparable to the obtained for the pool, being the analysis referring to the protein content and enzymatic activity in progress.

## References

[B1] Castro AM Pereira N Jr Produção, Propriedades e Aplicação de Celulases na Hidrólise de Resíduos Agroindustriais Química Nova 2010 33 1 181 188

[B2] Wakiyama M Tanaka H Yoshihara K Hayashi S Ohta K Purification and Properties of Family-10 Endo-1,4-β-Xylanase from Penicillium citrinum and Structural Organization of Encoding Gene Journal of Bioscience and Bioengineering 2008 105 4 367 374 10.1263/jbb.105.367 18499053

[B3] Ruegger MJS Tauk-Tornisielo SM Atividade da celulase de fungos isolados do solo da Estação Ecológica de Juréia-Itatins, São Paulo, Brasil Revista Brasileira de Botanica 2004 27 2 205 211

[B4] Szijártó N Szengyel Z Lidén G Réczey K Dynamics of Cellulase Production by Glucose Grown Cultures of Trichodermareesei Rut-C30 as a Response to Addition of Cellulose Applied Biochemistry and Biotechnology 2004 113 115 124 10.1385/ABAB:113:1-3:115 15054199

[B5] Ghose TK Measurement of Cellulase Activities Pure and Applied Chemistry 1987 59 2 257 268

